# The well-designed hierarchical structure of Musa basjoo for supercapacitors

**DOI:** 10.1038/srep20306

**Published:** 2016-02-04

**Authors:** Kaiwen Zheng, Xiaorong Fan, Yingzhu Mao, Jingkai Lin, Wenxuan Dai, Junying Zhang, Jue Cheng

**Affiliations:** 1 Key Laboratory of Carbon Fiber and Functional Polymers (Beijing University of Chemical Technology), Ministry of Education, Beijing 100029, China; 2 State Key Laboratory of Organic-Inorganic Composites, Beijing University of Chemical Technology, Beijing 100029, China; 3 College of Civil Engineering and Architecture, Zhejiang University, Hangzhou 310058, China; 4 Joint Research Centre for Biomedical Engineering, Department of Electronic Engineering, The Chinese University of Hong Kong, Hong Kong SAR, China

## Abstract

Application of biological structure is one of the hottest topics in the field of science and technology. The unimaginable and excellent architectures of living beings supporting their vital activities have attracted the interests of worldwide researchers. An intriguing example is Musa basjoo which belongs to the herb, while appears like a tree. The profound mystery of structure and potential application of Musa basjoo have not been probed. Here we show the finding of the hierarchical structure of Musa basjoo and the outstanding electrochemical performance of the super-capacitors fabricated through the simple carbonization of Musa basjoo followed by KOH activation. Musa basjoo has three layers of structure: nanometer-level, micrometer-level and millimeter-level. The nanometer-level structure constructs the micrometer-level structure, while the micrometer-level structure constructs the millimeter-level structure. Based on this hierarchical structure, Musa basjoo reduces the unnecessary weight and therefore supports its huge body. The super-capacitors derived from Musa basjoo display a high specific capacitance and a good cycling stability. This enlightening work opens a window for the applications of the natural structure and we hope that more and more people could pay attention to the bio-inspired materials.

In nature, most herbaceous plants are distinguished visibly from woody plants by their appearances as herbaceous plants are small and delicate, while woody plants are tall and strong. The primary cause leading to their disparate appearances is the structure and composition of their stems. Woody plants have cambium which makes them grow constantly (secondary growth), therefore becoming taller and thicker year by year, while herbaceous plants do not have such tissue and ability^1^. Thus, most herbaceous plants have succulent and soft stems which make them easy to lodging, and that is the reason why we call them “grass” and call woody plants “tree”.

However, there are exceptions in terms of this classification, such as Bamboo^2^ and Musa basjoo (Fig. 1a). They are two kinds of herbaceous plants but as tall and straight as trees. Bamboo is well known to have bamboo joints^3^ to support its weight, while the intriguing and less studied Musa basjoo doesn’t have such kind of structure. Curiosity drives us to figure out the reason for the unusual height of Musa basjoo.

## Results

### The millimeter-level structure of Musa basjoo

**Figure 1:**
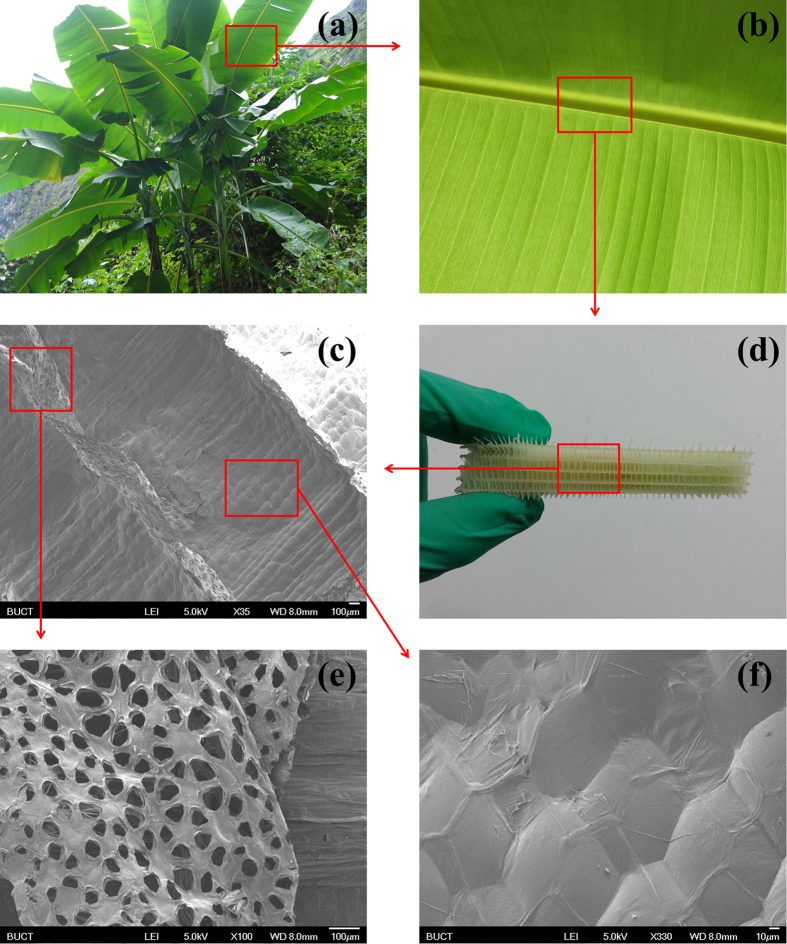
The photographs of Musa basjoo (**a**), stem and leaf vein (**b**), millimeter-level pores (**d**); and the SEM images of millimeter-level pores (**c**), flower-like micrometer-level pores (**e**) on the pore walls of millimeter-level pores, balloon-like micrometer-level spheres (**f**) on the pore floors of millimeter-level pores.

To find the answer, some fresh Musa basjoo was collected. After that, we stripped away the epidermis of stem and leaf vein (Fig. 1b), which support the weight of Musa basjoo. Interestingly, inside the stem and leaf vein, there is fence-like 3D porous network structure (the pore size is about a few millimeters), which is visible to the naked eyes (Fig. 1d). The fence-like 3D networks constructed by millimeter-level pores are differ from the structure of any woody plants, as they dramatically reduce the weight of Musa basjoo; and they are also differ from the structure of normal herbaceous plants, as they are well-designed, even-loaded and able to support the huge body of Musa basjoo.

### The micrometer-level structure of Musa basjoo

**Figure 2:**
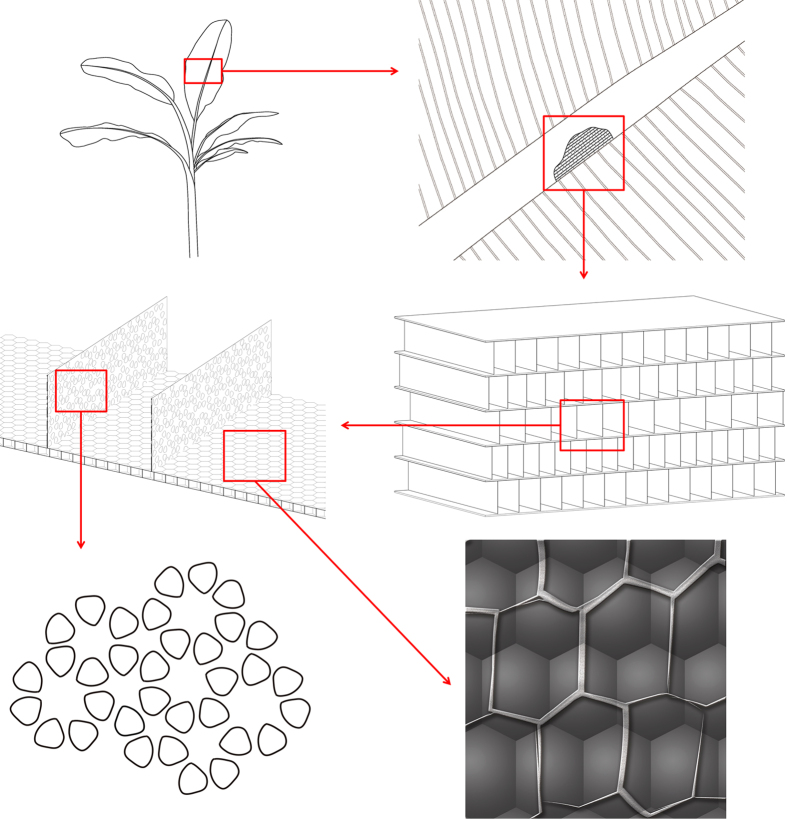
The corresponding abstract mathematical models of Figure 1.

**Figure 3:**
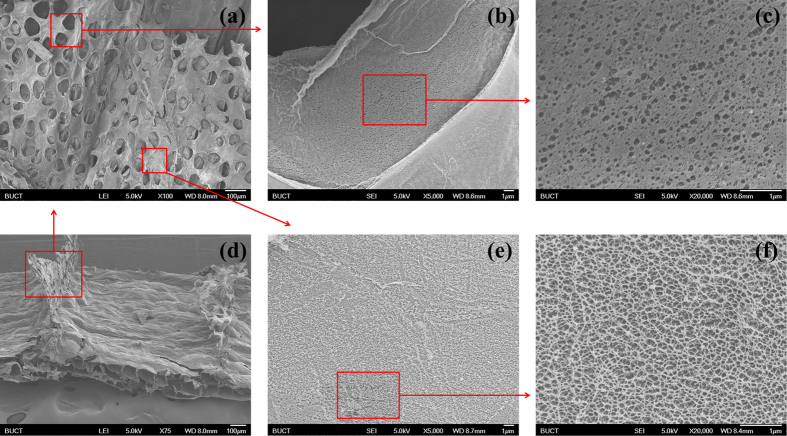
The SEM images of millimeter-level pores (**d**), flower-like micrometer-level pores (**a**), nanometer-level pores on the edge of “petal” (**b,c**) and the center of “flower” (**e,f**).

**Figure 4:**
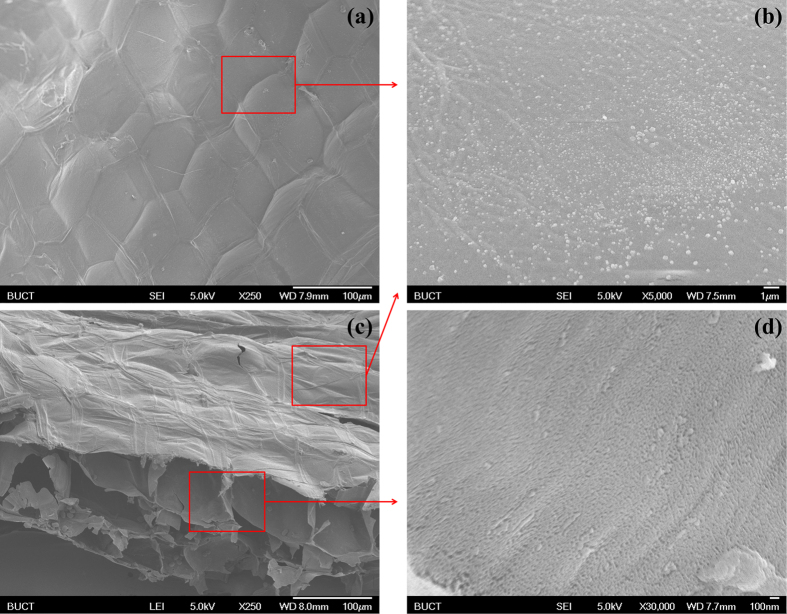
The SEM images of top view (**a**) and side view (**c**) of balloon-like micrometer-level spheres, papillae on the films of micrometer-level spheres (**b**) and nanometer-level pores on the skeletons of micrometer-level spheres (**d**).

Moreover, in light of the strong belief of nature^4^,^5^,^6^,^7^,^8^, we conjecture there will be better-designed and more elaborate micro-structure inside the millimeter-level structure. To study the micro-structure of Musa basjoo, we turned its fresh tissues into aerogel by freeze-drying, and observed them under scanning electron microscope (SEM). The SEM images of Musa basjoo and their corresponding abstract mathematical models are shown in Figs. 1, 2, 3 and 4. We were surprised to find out that there is unique micro-structure on the pore walls and pore floors of the millimeter-level pores (Fig. 1c). At low magnifications (×35 to ×75), we first found that there are micrometer-level pores (Fig. 1c, about 50 μm) on pore walls of the millimeter-level pores and neat micrometer-level spheres (Fig. 1c, about 100 μm) on pore floors of the millimeter-level pores. Further magnifying (×100), we were amazed by the finding that the micrometer-level pores present a flower-like structure (5, 6, 7 or 8 petals) instead of random arrangement (Fig. 1e). And this flower-like structure has been proved to be a well-designed structure with excellent mechanical properties, and there are other natural species and manmade devices tailored in the similar structure, such as lotus root, snowflake^9^, cosmos and the wheel hub of tire. In this structure, each “petal” (micrometer-level pore) is the component of the three surrounding “flowers”, that is, one “petal” is shared by three “flowers”, and this phenomenon is called “Archimedean tiling” in mathematics^10^. Moreover, at high magnifications (×250 to × 330), the micrometer-level spheres, properly speaking, aren’t globular but hexagonal (Fig. 1f). As is known to all, the hexagonal grid (hexagonal tiling, hexagonal lattice) has been proved to be a very stable and material-saving structure and there are many other examples in nature, such as honeycomb, photonic crystal^11^,^12^ and graphene^13^. The Chinese national aquatics center (Water Cube, the main swimming pool of the 2008 Beijing Olympic Games) is also of the same kind of design. Absorbingly, there is another hexagonal grid (constituted by fasciculus) on the surface of the previous mentioned hexagonal grid (constituted by micrometer-level spheres), while these two hexagonal grids are arranged alternately to reinforce each other (Fig. 1f). What’s more, it can be seen from the cross sections that the micrometer-level spheres are hollow (Fig. 3d), that is, they are constructed by skeleton and film which play supporting and covering roles, respectively. This balloon-like hollow structure can further reduce the weight of Musa basjoo.

### The nanometer-level structure of Musa basjoo

Further magnifying (×5000 to × 20000), we found a large number of nanometer-level pores (Fig. 3, about 100 nm) on the edge of “petal” and the center of “flower” of the flower-like micrometer-level pores. In other words, the skeletons of the flower-like micrometer-level pores look solid, but in fact they’re cribrate and constructed by nanometer-level pores. Amusingly, the pore walls of the millimeter-level pores look solid but are constructed by micrometer-level pores, while the skeletons of the micrometer-level pores look solid but are constructed by nanometer-level pores. Similarly, the pore floors of the millimeter-level pores look solid but are constructed by hollow micrometer-level spheres, while the skeletons of the micrometer-level spheres look solid but are constructed by nanometer-level pores as well (Fig. 4). And there are other examples in nature in terms of this amazing phenomenon, such as solid macroscopic object made by hollow microscopic atoms. Moreover, we have also found many papillae (about a few hundred nm) on the films of micrometer-level spheres (Fig. 4), like lotus leaf^14^,^15^. Herein, we forecast that this structure may also have super-hydrophobic and self-cleaning effect^16^, which prevent the plant from flooding when the rainwater enters from the break.

## Discussion

**Figure 5:**
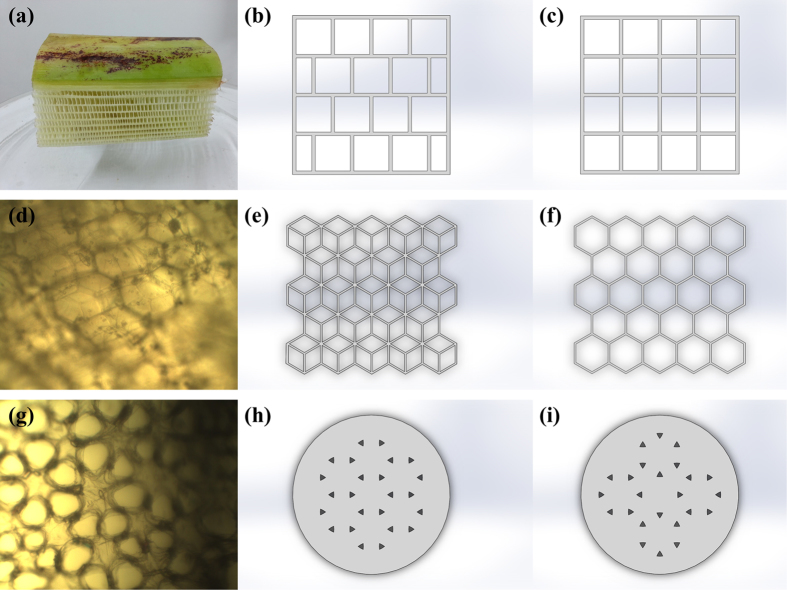
The photograph of millimeter-level pores (**a**); the optical microscope images of micrometer-level spheres (**d**) and micrometer-level pores (**g**); the abstract models of millimeter-level pores (**b**), micrometer-level spheres (**e**) and micrometer-level pores (**h**); and the contrast models of millimeter-level pores (**c**), micrometer-level spheres (**f**) and micrometer-level pores (**i**).

**Figure 6:**
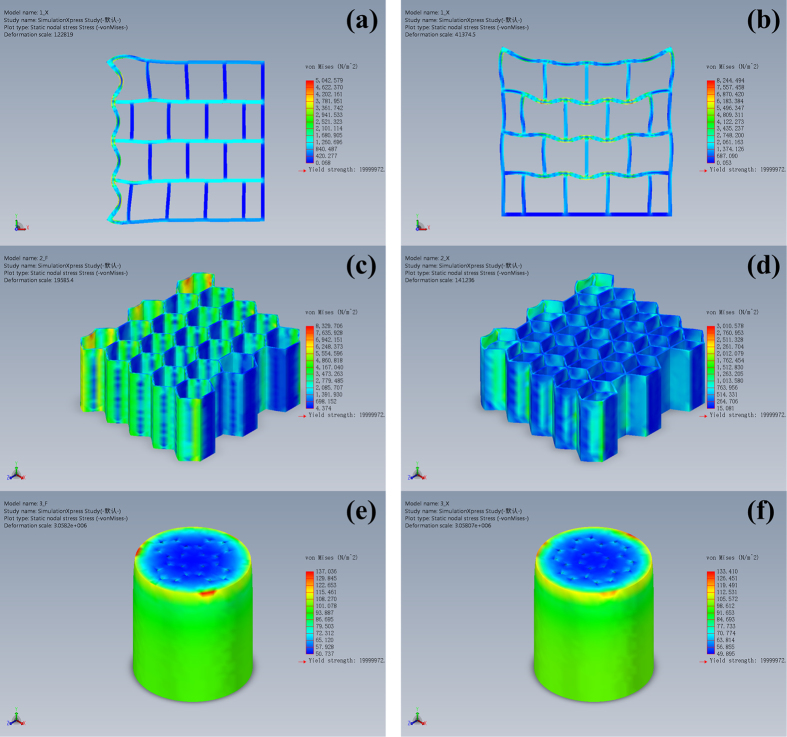
The stress nephograms of the contrast models of micrometer-level spheres (**c**) and micrometer-level pores (**e**); and the abstract models of millimeter-level pores (**a,b**), micrometer-level spheres (**d**) and micrometer-level pores (**f**).

**Figure 7:**
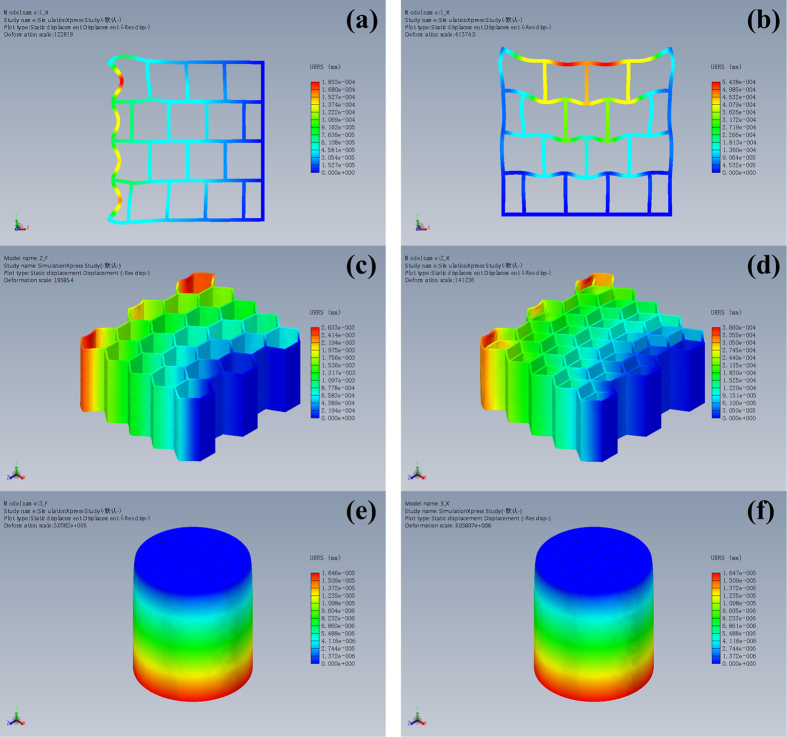
The displacement nephograms of the contrast models of micrometer-level spheres (**c**) and micrometer-level pores (**e**); and the abstract models of millimeter-level pores (**a,b**), micrometer-level spheres (**d**) and micrometer-level pores (**f**).

To further quantitatively analyze the hierarchical structure of Musa basjoo, we use mechanical simulation analysis (SolidWorks) to study the abstract models and contrast models of the fence-like millimeter-level structure, honeycomb-like micrometer-level spheres and flower-like micrometer-level pores of Musa basjoo. Their photographs, abstract models and contrast models are shown in Fig. 5. The stress and displacement nephograms are given in Figs 6 and 7, respectively. It can be clearly observed from Fig. 5a–c that comparing to perfectly symmetrical contrast model, the photograph and the abstract model of millimeter-level structure are staggered. Specifically speaking, in X direction (gravity direction), the millimeter-level structure has “straight paths” from top to bottom, so that gravity can be transmitted along those paths straightly down. Hence, the structure in X direction has a more concentrated stress and a smaller strain (Figs 6a and 7a), which shows the characteristics of rigid material. This design helps the plant maintain a constant height and bear the weight. At the same time, in Y direction (vertical to the gravity direction), the millimeter-level structure does not have those kind of “straight paths”, so that the impact force has to decompose and compose between each layer instead of being transmitted from side to side straightly. Thus, the structure in Y direction has a more dispersed stress and a larger strain (Figs 6b and 7b), which behaves like an elastic material. This design can absorb impact through deformation, therefore prevents the plant from lodging under storm condition. Similarly, comparing to contrast model (single hexagonal grid, Figs 6c and 7c), the abstract model (double hexagonal grids, Figs 6d and 7d) of the micrometer-level spheres has a lower maximum stress and strain, as well as a more uniform stress distribution under the same material, load and hollow area, therefore proving the interlaced double hexagonal grids do reinforce each other. Likewise, Figs 6e,f, and 7e,f show that the abstract model (flowers of pairwise correlation) of the flower-like micrometer-level pores has a better mechanical property than the contrast model (flowers of mutual independence) under the same material, load, number of petals and hollow area. However, the nanometer-level structure of Musa basjoo is too complex to draw the abstract model of it, therefore we use carbonization process rather than mechanical simulation analysis to study its mechanical property.

In summary, the stem of herbaceous plant is hollow while the woody plant is solid, and the stem of Musa basjoo is half-solid. The nanometer-level structure of Musa basjoo constructs its micrometer-level structure, while the micrometer-level structure constructs its millimeter-level structure. Based on this hierarchical structure, Musa basjoo reduces its unnecessary weight and decreases its density. And through well-designed structure, the stem and leaf vein still have good mechanical property under hollow condition to support the huge plant. Thus, Musa basjoo, one kind of herbaceous plants without bamboo-joint-like structure, can grow as tall and thick as a tree. Herein, we are once again amazed by the workmanship of nature.

After making clear the morphology and structure of Musa basjoo and the possible function they serve, we start to consider about their potential applications. There has been a world-wide focus on bio-inspired materials^17^,^18^,^19^,^20^,^21^,^22^, and these inventions have played an important role on the progress of society, such as radar (inspired by bat^23^,^24^), sonar (inspired by dolphin^25^,^26^), self-clearing clothes (inspired by lotus leaf) and so on. Here are some potential applications of the structure of Musa basjoo. On one hand, the nanometer-level pores are appropriate for being the carbon source of carbon electrodes and super-capacitors^27^,^28^, the template for fabricating novel nano-materials^29^ and the support of catalyst^30^. On the other hand, the well-designed structure of flower-like micrometer-level pores and honeycomb-like micrometer-level spheres is inspiring, the architect may design beautiful and material-saving building with high mechanical property accordingly. Furthermore, this hierarchical network system can be used for filtration, separation and purification.

**Figure 8:**
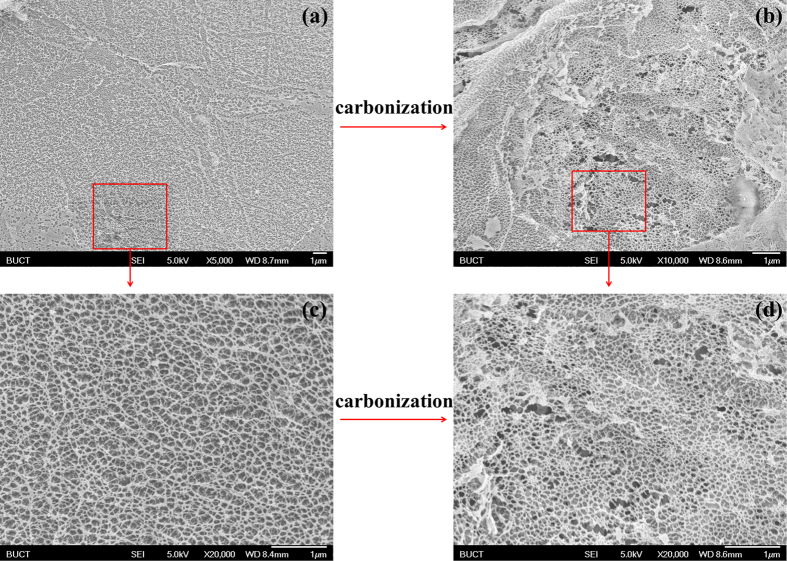
The SEM images of nanometer-level pores before (**a,c**) and after (**b,d**) carbonization.

Among those potential applications of the structure of Musa basjoo, being the carbon source of super-capacitors might be the most suitable one, as this porous network system has large specific surface area and high porosity. Moreover, electrons and ions can nearly reach every corner of the super-capacitors with the help of the interconnected hierarchical structure, like the arteries-capillaries-veins system in our body. Furthermore, most nanometer-level pores still exist after carbonization and only few of them collapsed during the process (Fig. 8), which shows the good mechanical property of this structure.

**Figure 9:**
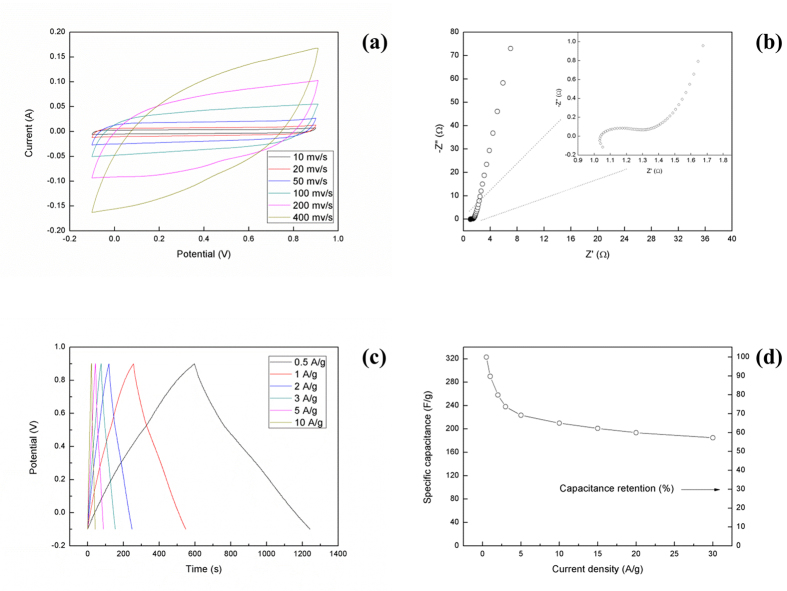
The electrochemical properties of the supercapacitors prepared from Musa basjoo: (**a**) CV curves at different scan rates; (**b**) Nyquist plots with the inset showing the high frequency region; (**c**) Charge/discharge curves at different current densities; (**d**) Specific capacitance at different current densities.

**Figure 10:**
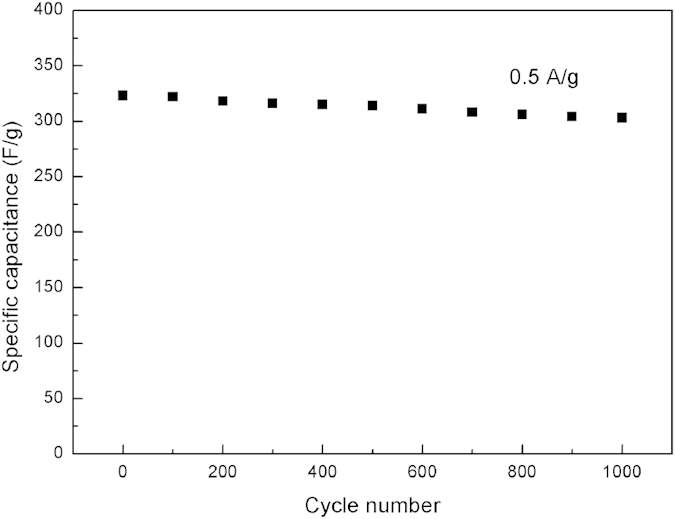
The cycling stability of the supercapacitors prepared from Musa basjoo at a current density of 0.5 A/g.

Here we show our attempt in fabricating super-capacitors by using the nanometer-level structure of Musa basjoo. As we can see from Fig. 9, the carbon prepared from Musa basjoo does show excellent electrochemical performance by a high specific capacitance of 323 F/g at a current density of 0.5 A/g within a potential window of −0.1 to 0.9 V in 1 M H_2_SO_4_ solution. At the same time, the resultant super-capacitors also exhibit good cycling stability with 94% of the capacitance retention after 1000 cycles of charge/discharge (Fig. 10).

This enlightening work might open a window for the applications of natural structure and we hope that more and more people could pay attention to bio-inspired materials.

## Methods

For the synthesis of the super-capacitors from Musa basjoo, we turned the fresh tissue of Musa basjoo into aerogel by freeze-drying (−65 °C, 24 h). And the aerogel was pre-carbonized at 450 °C for 1.5 h with a heating rate of 5 °C/min under a nitrogen atmosphere. Then, the black product was mixed evenly with 0.1 M KOH aqueous solution (the weight ratio of KOH and carbon is 1.5: 1). Subsequently, the black mixture was dried at 85 °C for 5 h to remove water and make sure that KOH was mixed thoroughly with carbon. After that, the resulting mixture was placed in a crucible with a heating rate of 5 °C/min under a nitrogen atmosphere by the following procedure: firstly, the mixture was heated to 500 °C and hold for 0.5 h; afterwards, the temperature was raised to 700 °C and kept for another 0.5 h; Lastly, the temperature was increased to 800 °C and hold for 1 h. Finally, the resultant powder was washed by 0.1 M HCl aqueous solution to remove residual inorganic impurities, and then washed by distilled water until pH reached 7.

## Additional Information

**How to cite this article**: Zheng, K. *et al.* The well-designed hierarchical structure of Musa basjoo for supercapacitors. *Sci. Rep.* **6**, 20306; doi: 10.1038/srep20306 (2016).
